# Combined inhibition of PIM and CDK4/6 suppresses both mTOR signaling and Rb phosphorylation and potentiates PI3K inhibition in cancer cells

**DOI:** 10.18632/oncotarget.27539

**Published:** 2020-04-28

**Authors:** Lacey M. Litchfield, Karsten Boehnke, Manisha Brahmachary, Cecilia Mur, Chen Bi, Jennifer R. Stephens, J. Michael Sauder, Sonia M. Gutiérrez, Ann M. McNulty, Xiang S. Ye, Wenjuan Wu, María José Lallena, Xueqian Gong, Farhana F. Merzoug, Valerie M. Jansen, Sean G. Buchanan

**Affiliations:** ^1^Eli Lilly and Company, Indianapolis, IN, USA; ^2^Eli Lilly and Company, New York, NY, USA; ^3^Eli Lilly and Company, Alcobendas, Madrid, Spain; ^4^Eli Lilly and Company, San Diego, CA, USA; ^5^Eli Lilly and Company, Shanghai, China

**Keywords:** abemaciclib, CDK4/6, PIM, mTOR, S6

## Abstract

Aberrant activation of mitogenic signaling pathways in cancer promotes growth and proliferation of cells by activating mTOR and S6 phosphorylation, and D-cyclin kinases and Rb phosphorylation, respectively. Correspondingly, inhibition of phosphorylation of both Rb and S6 is required for robust anti-tumor efficacy of drugs that inhibit cell signaling. The best-established mechanism of mTOR activation in cancer is via PI3K/Akt signaling, but mTOR activity can also be stimulated by CDK4 and PIM kinases. In this study, we show that the CDK4/6 inhibitor abemaciclib inhibits PIM kinase and S6 phosphorylation in cancer cells and concurrent inhibition of PIM, CDK4, and CDK6 suppresses both S6 and Rb phosphorylation. *TSC2* or *PIK3CA* mutations obviate the requirement for PIM kinase and circumvent the inhibition of S6 phosphorylation by abemaciclib. Combination with a PI3K inhibitor restored suppression of S6 phosphorylation and synergized to curtail cell growth. By combining abemaciclib with a PI3K inhibitor, three pathways (Akt, PIM, and CDK4) to mTOR activation are neutralized, suggesting a potential combination strategy for the treatment of *PIK3CA*-mutant ER+ breast cancer.

## INTRODUCTION

Cyclin-dependent kinases (CDK) 4 and 6, which phosphorylate the retinoblastoma tumor suppressor protein Rb to promote G1 to S transition, are commonly dysregulated in cancer [1]. Genetic alterations that increase expression or stability of D-cyclins result in activation of D-cyclin dependent kinases, and, thus, confer dependence on CDK4/6 for continued cell cycle progression and cell proliferation [2]. D-cyclin levels are also modulated downstream of mitogen and hormone signaling pathways, including PI3K, MAPK, and ER [3–5], suggesting that aberrant activation of these pathways may also result in CDK4/6 dependencies that could be exploited clinically. In addition to stimulating cell cycle initiation via D-cyclin dependent kinases and Rb phosphorylation, these signaling pathways promote cell growth via mTOR activation and S6 phosphorylation, and it has been shown that inhibition of phosphorylation of both Rb and S6 is required for robust anti-tumor efficacy of drugs that inhibit cell signaling [6–12]. mTOR activity can also be stimulated, independently of the PI3K pathway, by CDK4 and PIM kinases [13–16], both of which have been identified as potential mechanisms of resistance to PI3K inhibitors in breast cancer [6, 15].

The CDK4/6 inhibitors abemaciclib, palbociclib, and ribociclib have emerged as important new treatment options for HR+, HER2- advanced breast cancer in combination with endocrine therapy and are under investigation for additional indications [17–19]. Several distinguishing characteristics of the three drugs have been reported. Abemaciclib is structurally distinct from palbociclib and ribociclib. In addition, abemaciclib has greater potency against CDK4 than CDK6 in enzymatic assays [20]. Clinically, lower rates of neutropenia result from treatment with abemaciclib allow for continuous, twice-daily dosing; palbociclib and ribociclib are administered on an intermittent dosing schedule [1, 21]. Abemaciclib has also demonstrated single-agent activity [21, 22] and is, uniquely among the CDK4/6 inhibitors, approved by the FDA for use as monotherapy [23], but the mechanistic basis for this activity remains to be fully understood. The approved CDK4/6 inhibitors show activity against additional kinases in *in vitro* assays [24–27], but, for these additional targets of the drugs, direct evidence of inhibition in cells is limited, and in most cases it is unlikely that they are potently inhibited in cells at the plasma concentrations achieved at clinical doses [2, 28].

Here we show that abemaciclib can suppress the kinase activity of the oncoprotein PIM, and that, similar to PIM inhibitors, abemaciclib inhibits S6 phosphorylation in cells with wild-type *PIK3CA* and *TSC2*. Additionally, we evaluate the potential utility of concurrent treatment with abemaciclib and the PI3K inhibitor BYL719 (alpelisib) in *PIK3CA* mutant breast cancer. Our results suggest that abemaciclib can inhibit the mTOR pathway independently of its effects on Rb and support combining abemaciclib with PI3K/mTOR pathway inhibitors to fully suppress phosphorylation of S6 via multiple inputs.

## RESULTS

### Single-agent abemaciclib rapidly inhibits mTOR signaling

**Figure 1:**
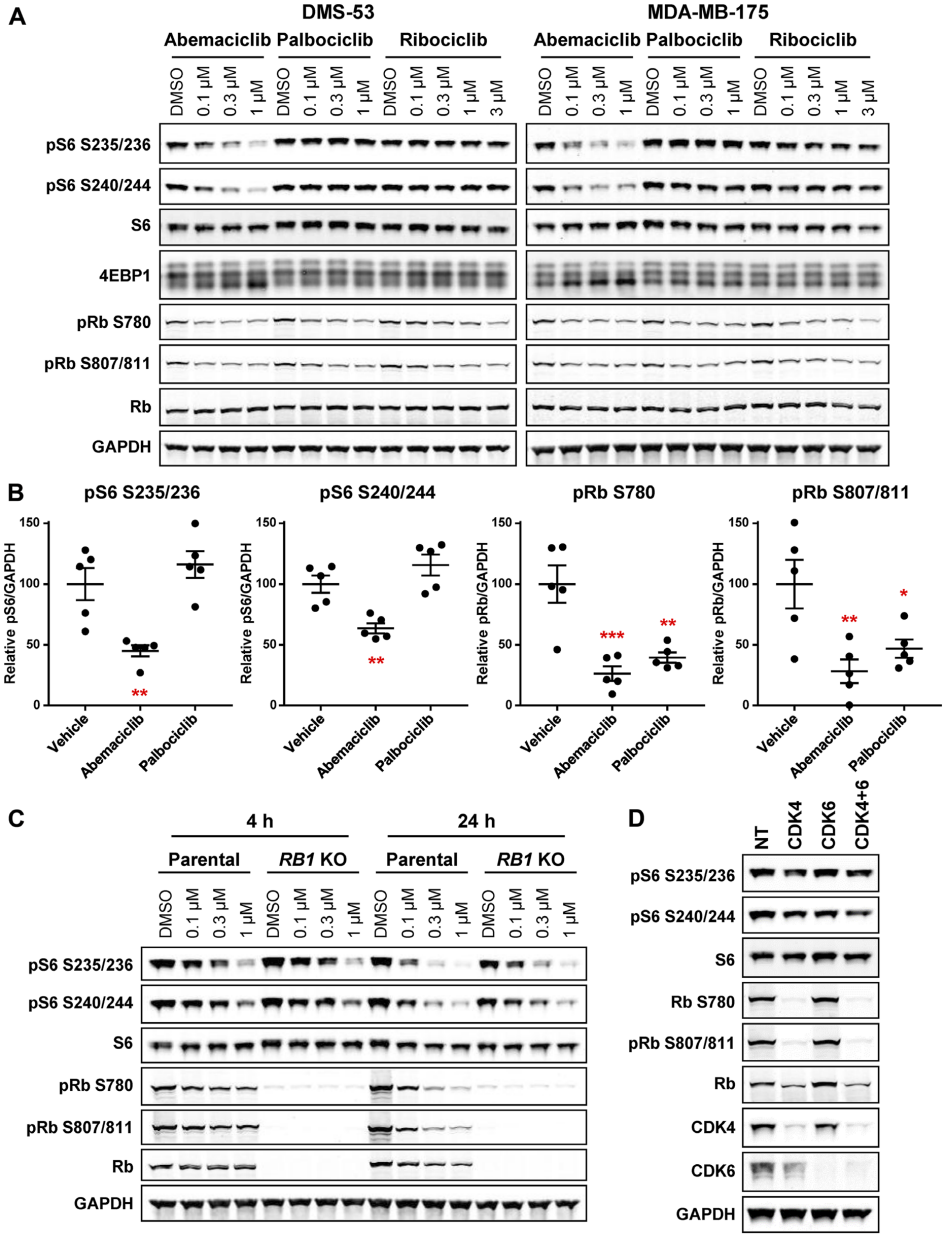
Abemaciclib inhibits S6 phosphorylation independent of effects on CDK4/6 and Rb. (**A**) DMS-53 and MDA-MB-175 cells were treated with the indicated concentrations of abemaciclib, palbociclib, or ribociclib for 4 h and analyzed by western blot. (**B**) Mice bearing A549 xenograft tumors were treated with a single dose of abemaciclib or palbociclib (50 mg/kg). Tumors were collected 24 h post-treatment and analyzed by western blot. Plots indicate mean ± SEM (*n* = 5/group), relative to vehicle control. ^*^ *p* < 0.05; ^**^ *p* < 0.01; ^***^ *p* < 0.001. (**C**) DMS-53 parental or *RB1* KO cells were treated with the indicated concentrations of abemaciclib for 4 or 24 h and analyzed by western blot. (**D**) DMS-53 cells were transfected with *CDK4*, *CDK6*, *CDK4*+*CDK6*, or non-targeting control (NT) siRNA for 48 h and analyzed by western blot.

While evaluating the effects of CDK4/6 inhibitors on growth and proliferative signaling pathways, we noticed that, intriguingly, abemaciclib treatment rapidly suppressed S6 and 4EBP1 phosphorylation in several cell lines, including DMS-53 small cell lung cancer (SCLC) and MDA-MB-175 breast cancer cells (Figure 1A, Supplementary Figure 1A, 1B). In the same cell lines, palbociclib and ribociclib did not alter S6 or 4EBP1 phosphorylation, although, as with abemaciclib, Rb phosphorylation was inhibited. In addition to DMS-53 and MDA-MB-175, inhibition of S6 phosphorylation was also observed following abemaciclib treatment in cell lines of several other cancer types, including mantle cell lymphoma (MCL; Jeko-1), pancreas cancer (MiaPaCa2), osteosarcoma (U2OS), melanoma (SK-MEL-28), non-small cell lung cancer (NSCLC; A549), and even Rb-null triple negative breast cancer (TNBC; MDA-MB-468) (Supplementary Figure 1C, 1D). The major metabolites of abemaciclib, M2 and M20 [28, 29], also inhibited S6 phosphorylation (Supplementary Figure 1E). *In vivo*, S6 phosphorylation was inhibited by abemaciclib, but not by palbociclib, in A549 xenograft tumors, while reduced Rb phosphorylation was evident following treatment with either drug (Figure 1B). Inhibition of S6 phosphorylation was also sustained following prolonged exposure to abemaciclib for two cell doublings, indicating that this response is durable (Supplementary Figure 1F). Long-term treatment with palbociclib did not substantially affect S6 phosphorylation, suggesting the persistent mTOR pathway suppression by abemaciclib may not have been simply an indirect consequence of cell cycle inhibition (Supplementary Figure 1F). Indeed, loss of Rb expression in DMS-53 did not alter the ability of abemaciclib to inhibit S6 phosphorylation following either 4 or 24 h treatment (Figure 1C). CDK4 and/or CDK6 knockdown also had only a modest effect on S6 phosphorylation, similar to the results with palbociclib and ribociclib treatment, suggesting the effects of abemaciclib on mTOR signaling may be mediated by a CDK4/6-Rb-independent mechanism (Figure 1D, Supplementary Figure 2A).

### PIM kinase inhibition phenocopies mTOR suppression by abemaciclib

**Figure 2:**
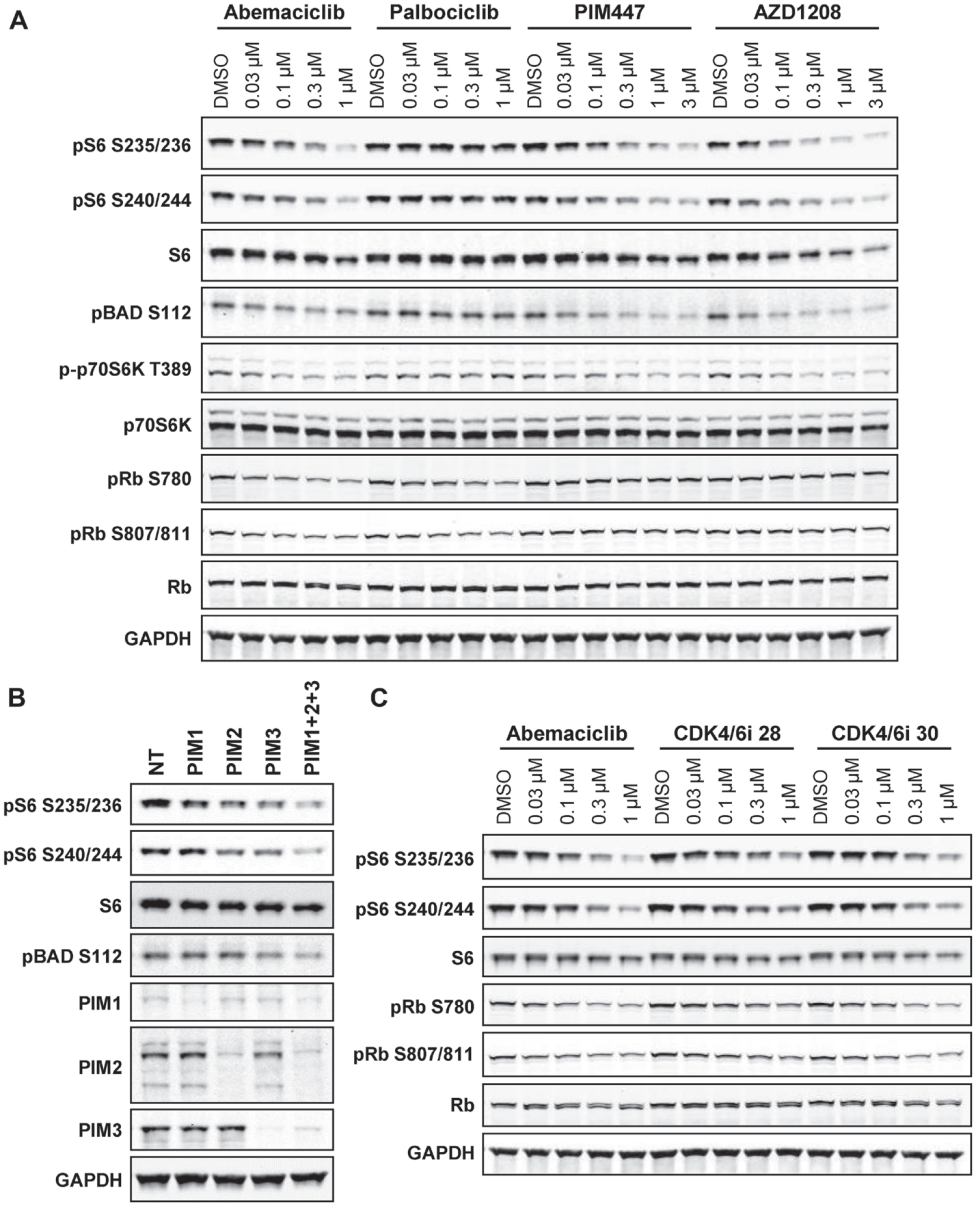
PIM kinase inhibition phenocopies effects of abemaciclib on mTOR signaling. (**A**) DMS-53 cells were treated with the indicated concentrations of abemaciclib, palbociclib, or PIM inhibitors PIM447 or AZD1208 for 4 h and analyzed by western blot. (**B**) DMS-53 cells were transfected with *PIM1*, *PIM2*, *PIM3*, *PIM1*+*PIM2*+*PIM3*, or non-targeting control (NT) siRNA for 48 h and analyzed by western blot. (**C**) DMS-53 cells were treated with the indicated concentrations of abemaciclib or two additional CDK4/6 inhibitors for 4 h and analyzed by western blot.

Another feature of abemaciclib is its reported inhibition of PIM kinases ([20], Supplementary Figure 2B). The PIM kinases, PIM1, PIM2, and PIM3, phosphorylate a diverse array of substrates regulating cell growth, and these substrates share significant overlap with known substrates of Akt signaling [16]. As potent effects of PIM inhibition on mTOR signaling (including S6 and p70S6K phosphorylation) have been reported [30, 31], we hypothesized that inhibition of PIM was a plausible explanation for the observed reduction in S6 phosphorylation by abemaciclib. Indeed, treatment with the PIM inhibitors PIM447 and AZD1208 closely phenocopied the ability of abemaciclib to inhibit phosphorylation of S6, p70S6K, and the PIM substrate BAD (Figure 2A). Knockdown of *PIM1*, *2*, and *3*, individually or in combination, likewise suppressed S6 and BAD phosphorylation (Figure 2B). Reduced phosphorylation of BAD was also evident following abemaciclib treatment *in vivo* (Supplementary Figure 2C). Inhibitors of DYRK1B (compound 33 [32]) or CDK9 (dinaciclib), additional kinases inhibited by abemaciclib in biochemical assays [20], did not impact S6 or p70S6K phosphorylation (Supplementary Figure 2D). Two additional CDK4/6 inhibitors identified during the development of abemaciclib, and closely related by chemical structure [33], likewise reduced phosphorylation of S6, p70S6K, and BAD, and were found to have activity against PIM kinases in biochemical and cellular assays (Figure 2C). Abemaciclib metabolites M2 and M20 were also found to inhibit PIM kinase (data not shown) consistent with their ability to inhibit S6 phosphorylation in cells (Supplementary Figure 1E).

A structural comparison of how abemaciclib and palbociclib could bind in the ATP pocket of the PIM1 isoform allowed us to understand their different activities. We modeled abemaciclib binding to the PIM1 catalytic site. Overlaying palbociclib on this model and inspection of the catalytic Lys67 (which salt-bridges Glu89 on the C-α-helix), gatekeeper (Leu120) and DFG domain showed multiple clashes which would prevent binding (Supplementary Figure 3). Abemaciclib, by contrast, is seen to (i) fill the ATP pocket under the p-loop, (ii) make hydrophobic contact with Val52, and (iii) make a favorable interaction with Lys67 explaining its potency against PIM kinase.

### Combined inhibition of CDK4/6 and PIM enhances suppression of mTOR and cell growth

**Figure 3:**
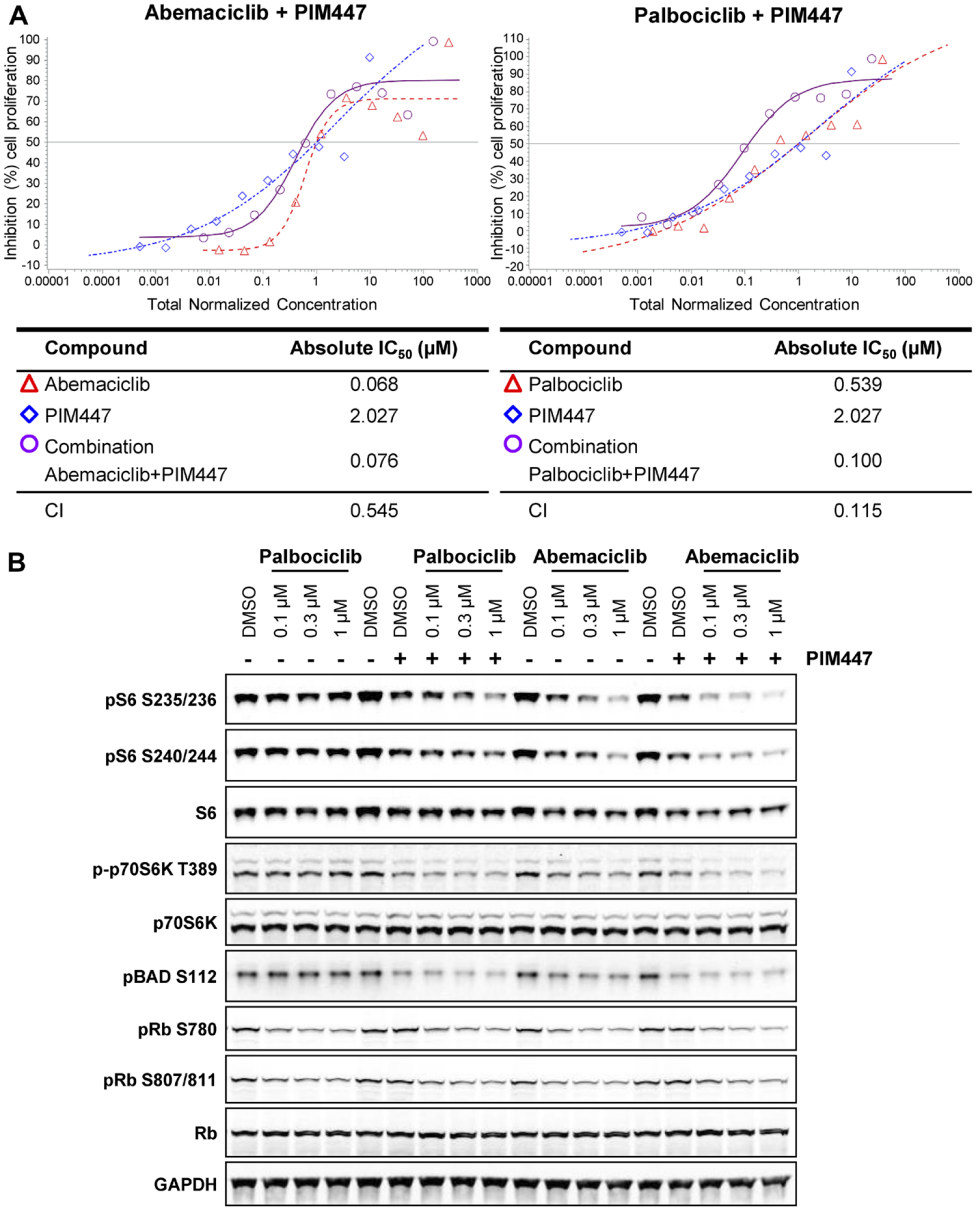
Combination CDK4/6 and PIM kinase inhibition suppresses mTOR signaling and cell growth. (**A**) DMS-53 cells were treated with the combination of PIM inhibitor PIM447 and abemaciclib or palbociclib for 2DT and cell growth was assessed by CellTiter-Glo. Curve shift analysis [66] was used to calculate a combination index (CI) as an indication of additivity or synergy between the compounds. (**B**) DMS-53 cells were treated with the combination of PIM inhibitor PIM447 (0.3 µM) and abemaciclib or palbociclib for 4 h and analyzed by western blot.

We next examined the impact of concurrent PIM and CDK4/6 inhibition by treating cells with PIM447 in combination with either abemaciclib or palbociclib. In DMS-53 cells abemaciclib monotherapy was approximately 8-fold more potent than palbociclib monotherapy (CTG IC_50_ 68 v 539 nM), despite similar inhibition of phospho-Rb (Figures 3A, 1A). The addition of PIM447 to palbociclib substantially improved potency to 100 nM, bringing it into the same range as abemaciclib monotherapy, and corresponding to significant synergy (combination index 0.115; 95% confidence interval 0.041–0.318) (Figure 3A). The PIM447/palbociclib combination also showed improved efficacy (% inhibition at the plateau of the dose response curve). Addition of PIM447 to abemaciclib, on the other hand, did not improve over abemaciclib’s monotherapy potency (IC_50_ 76 v 68 nM), and the combination was not synergistic (combination index 0.545; 95% confidence interval 0.124–1.786). The potency improvement conferred by addition of the PIM inhibitor to palbociclib is accompanied by inhibition of S6 phosphorylation to a degree comparable to abemaciclib monotherapy (Figure 3B).

### PIM regulation of mTOR signaling requires TSC2 and GSK3β

**Figure 4:**
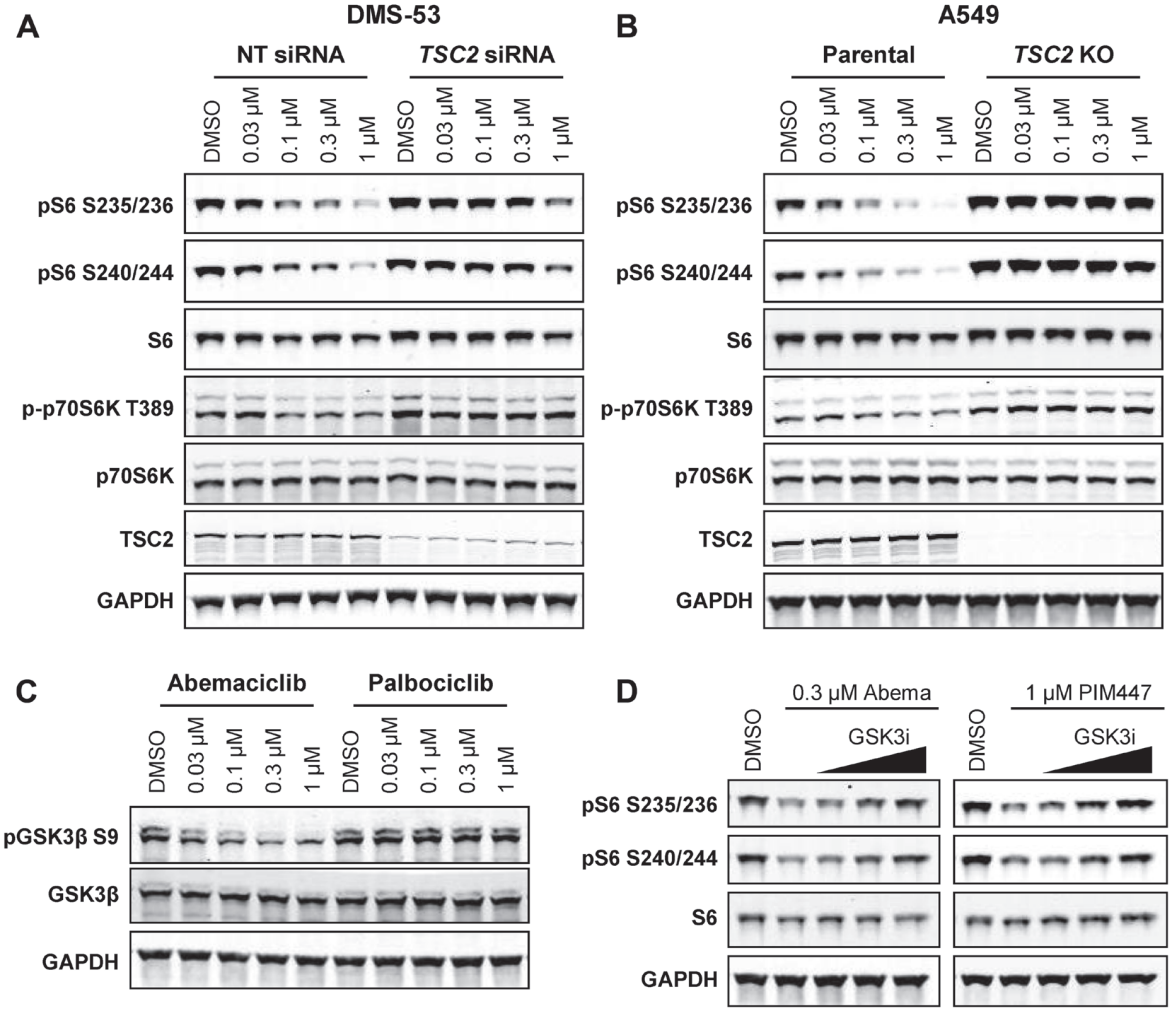
Inhibition of mTOR signaling by abemaciclib requires TSC2 and GSK3β. (**A**) DMS-53 cells were transfected with *TSC2* or non-targeting control (NT) siRNA for 48 h prior to treatment with abemaciclib for 4 h and analysis by western blot. (**B**) A549 parental or *TSC2* KO cells were treated and analyzed as in A. (**C**) DMS-53 cells were treated with the indicated concentrations of abemaciclib or palbociclib for 4 h and analyzed by western blot. (**D**) DMS-53 cells were treated with abemaciclib or PIM447, alone or in combination with increasing concentrations of GSK3 inhibitor LY2090314 (0.005, 0.05, or 0.5 µM), for 4 h and analyzed by western blot.

Several mechanisms have been postulated to underlie the control of mTOR signaling by PIM kinases, including phosphorylation and inactivation of mTOR suppressors PRAS40 and tuberin (TSC2) [34, 35]. PRAS40 phosphorylation was unchanged following treatment with abemaciclib or PIM inhibitors (Supplementary Figure 4), consistent with a previous report [35]. *TSC2* knockdown in DMS-53 (Figure 4A), or knockout in A549 (Figure 4B), prevented the reduction in S6 phosphorylation by abemaciclib, but not by the mTOR inhibitor everolimus (Supplementary Figure 5A, 5B), suggesting that PIM acts upstream of TSC2, although we have not been able to detect phosphorylation of TSC2 at a reported PIM-specific site (Ser1798 [35]) in DMS-53 cells (data not shown). Similarly, in SNU-886, a hepatocellular carcinoma cell line with natural *TSC2* loss, abemaciclib was unable to suppress S6 phosphorylation (Supplementary Figure 5C).

In addition to phosphorylation of TSC2, PIM was also reported to phosphorylate glycogen synthase kinase 3β (GSK3β) at Ser9, resulting in its inactivation [36]. As GSK3β has also been shown to phosphorylate and activate TSC2 to suppress mTOR signaling [37], indirect TSC2-dependent mTOR regulation by PIM could also occur. Indeed, phosphorylation of GSK3β at Ser9 was reduced following treatment with abemaciclib (but not palbociclib; Figure 4C), indicative of GSK3β activation, and addition of the GSK3 inhibitor LY2090314 [38] to abemaciclib or PIM447 reversed the inhibition of S6 phosphorylation by either drug (Figure 4D). These results define GSK3β suppression as a target of PIM responsible for its ability to activate the mTOR pathway and GSK3β activation as a key mechanism of inhibition of mTOR by abemaciclib and PIM kinase inhibitors.

### PI3K activity compensates for PIM in *PIK3CA* mutant breast cancer

**Figure 5:**
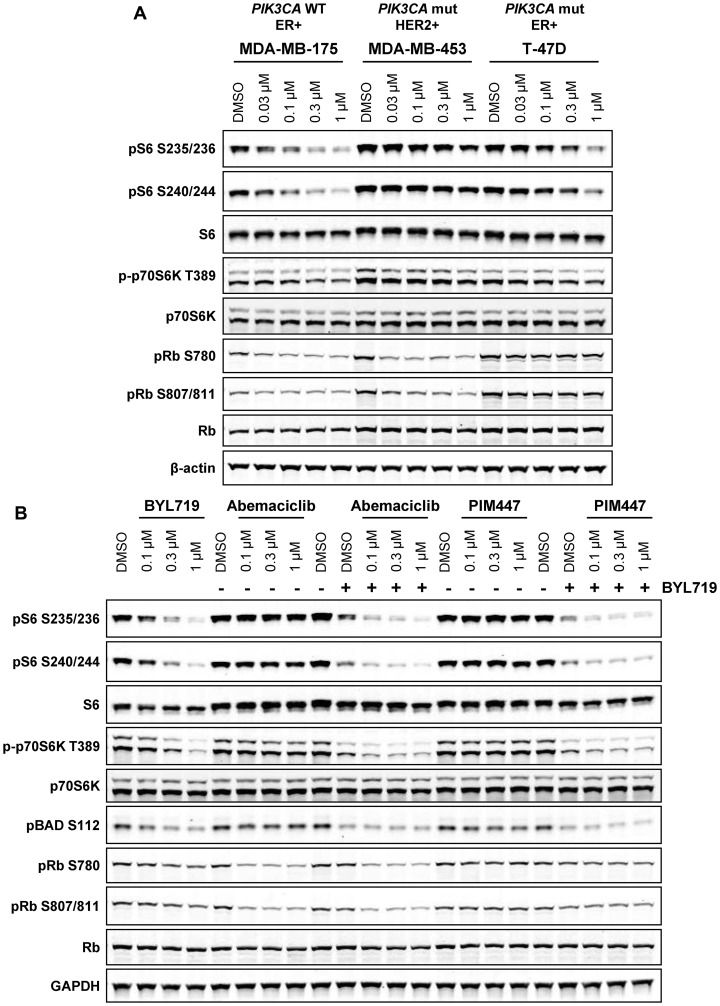
PI3K activity compensates for PIM inhibition in *PIK3CA* mutant breast cancer. (**A**) MDA-MB-175 (*PIK3CA* wt), MDA-MB-453 (*PIK3CA* mutant, HER2+), and T-47D (*PIK3CA* mutant) cells were treated with the indicated concentrations of abemaciclib for 4 h and analyzed by western blot. (**B**) MDA-MB-453 cells were treated with the combination of PI3K inhibitor BYL719 (0.3 µM) and abemaciclib or PIM447 for 4 h and analyzed by western blot.

Given the overlap between PIM and PI3K/Akt signaling and the identification of PIM kinases as mediators of resistance to PI3K and HER2 inhibition in breast cancer [15, 39], we next asked whether PI3K signaling in cell lines with *PIK3CA* and/or HER2 alterations would compensate for PIM. Inhibition of S6 and p70S6K phosphorylation by abemaciclib was substantially reduced in *PIK3CA* mut/HER2+ MDA-MB-453 and *PIK3CA* mut T-47D, as compared to *PIK3CA* WT MDA-MB-175 (Figure 5A). While single-agent treatment with abemaciclib or PIM447 was unable to inhibit downstream mTOR signaling in MDA-MB-453, concurrent treatment with the PI3K inhibitor BYL719 and either drug resulted in inhibition of S6 and p70S6K phosphorylation (Figure 5B). Intriguingly, this inhibition was superior to the inhibition by BYL719 alone. Together, these data suggest that the functional redundancy of PIM and PI3K/Akt signaling could be targeted by combination therapy to counteract compensatory signaling and augment the responses achieved by single-agent treatment.

### Concurrent abemaciclib and BYL719 treatment synergistically inhibits cell growth

**Figure 6:**
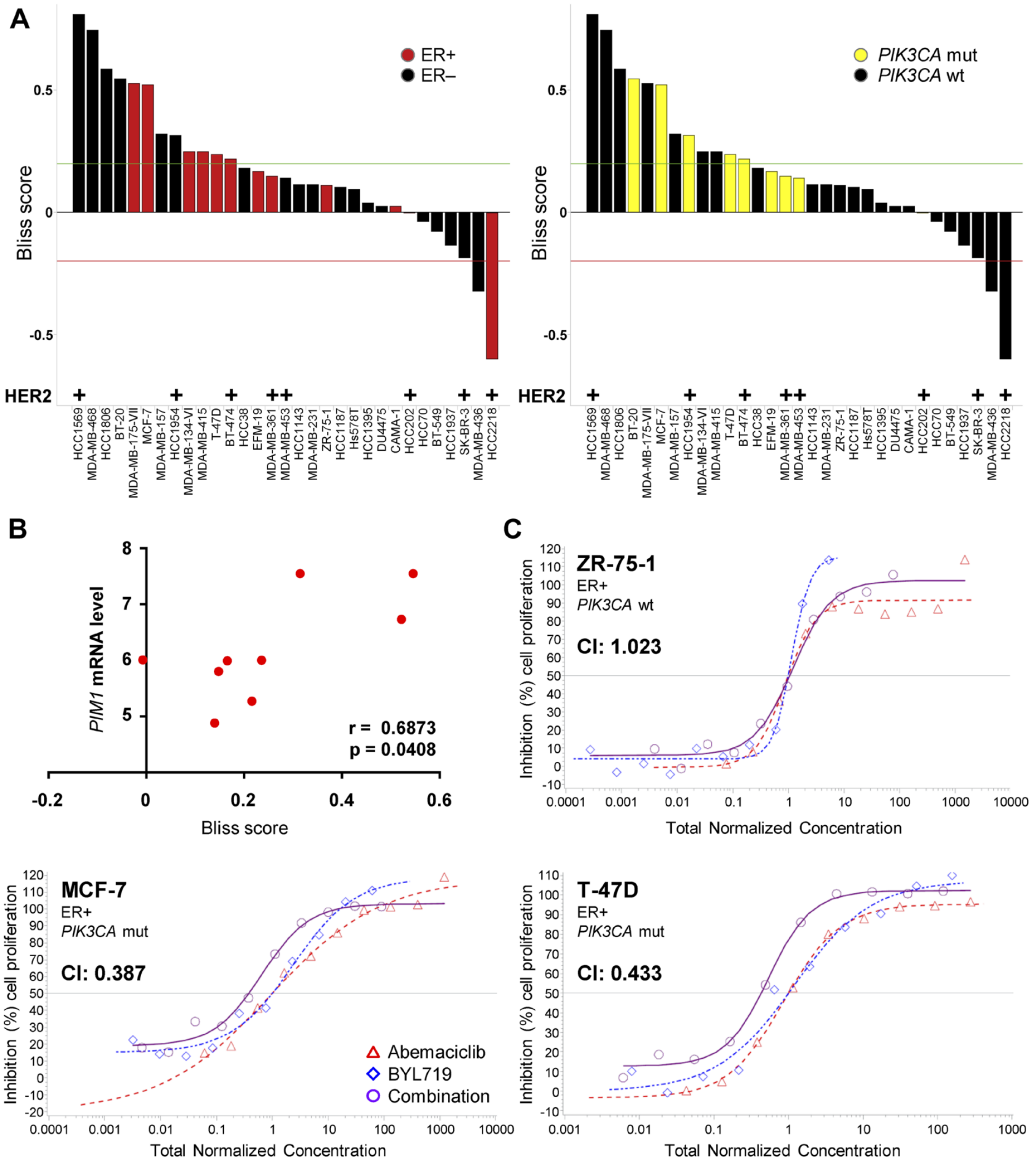
Combination treatment with abemaciclib and BYL719 synergistically inhibits breast cancer cell growth. (**A**) A panel of 31 breast cancer cell lines was treated with the combination of abemaciclib and PI3K inhibitor BYL719 for 2DT and cell growth was assessed by PI staining. ER expression/*PIK3CA* mutation status are displayed. (**B**) Correlation of *PIM1* mRNA levels (Cancer Cell Line Encyclopedia (CCLE) [68]) and abemaciclib-BYL719 Bliss score. (**C**) Confirmatory assays of MCF-7 (*PIK3CA* mutant), T-47D (*PIK3CA* mutant), or ZR-75-1 (*PIK3CA* wt) cells treated with the combination of abemaciclib and BYL719 for 2DT and assessed by PI staining. Curve shift analysis was used to calculate a combination index (CI) as an indication of additivity or synergy between the compounds.

To further explore the potential benefit of combining abemaciclib with PI3K inhibition, we tested the ability of concurrent treatment with abemaciclib and BYL719 to inhibit cell growth in panel of 31 breast cancer cell lines. The tested cell lines displayed a range of sensitivities to single-agent abemaciclib or BYL719 (Supplementary Figure 6A, 6B). Combination treatment resulted in synergistic inhibition of cell growth in twelve cell lines (Figure 6A) and was synergistic or additive in all 9 *PIK3CA* mut cell lines tested. Additionally, *PIM1* expression levels in *PIK3CA* mut cell lines correlated with the abemaciclib-BYL719 combination synergy score (Figure 6B). Confirmatory assays demonstrated synergism between abemaciclib and BYL719 in *PIK3CA* mut MCF-7 and T-47D cells, while the combination was additive in *PIK3CA* WT ZR-75-1 (Figure 6C). Similarly, the combination of a PDPK1 inhibitor (GSK2334470) and abemaciclib also synergistically inhibited growth of T-47D cells (Supplementary Figure 7A) and improved inhibition of downstream S6 and p70S6K phosphorylation (Supplementary Figure 7B), suggesting the observed effects of targeting PI3K in combination with abemaciclib may also apply to drugs targeting other nodes of the pathway [40].

### mTOR signaling is suppressed in breast cancer patients following abemaciclib treatment

**Figure 7:**
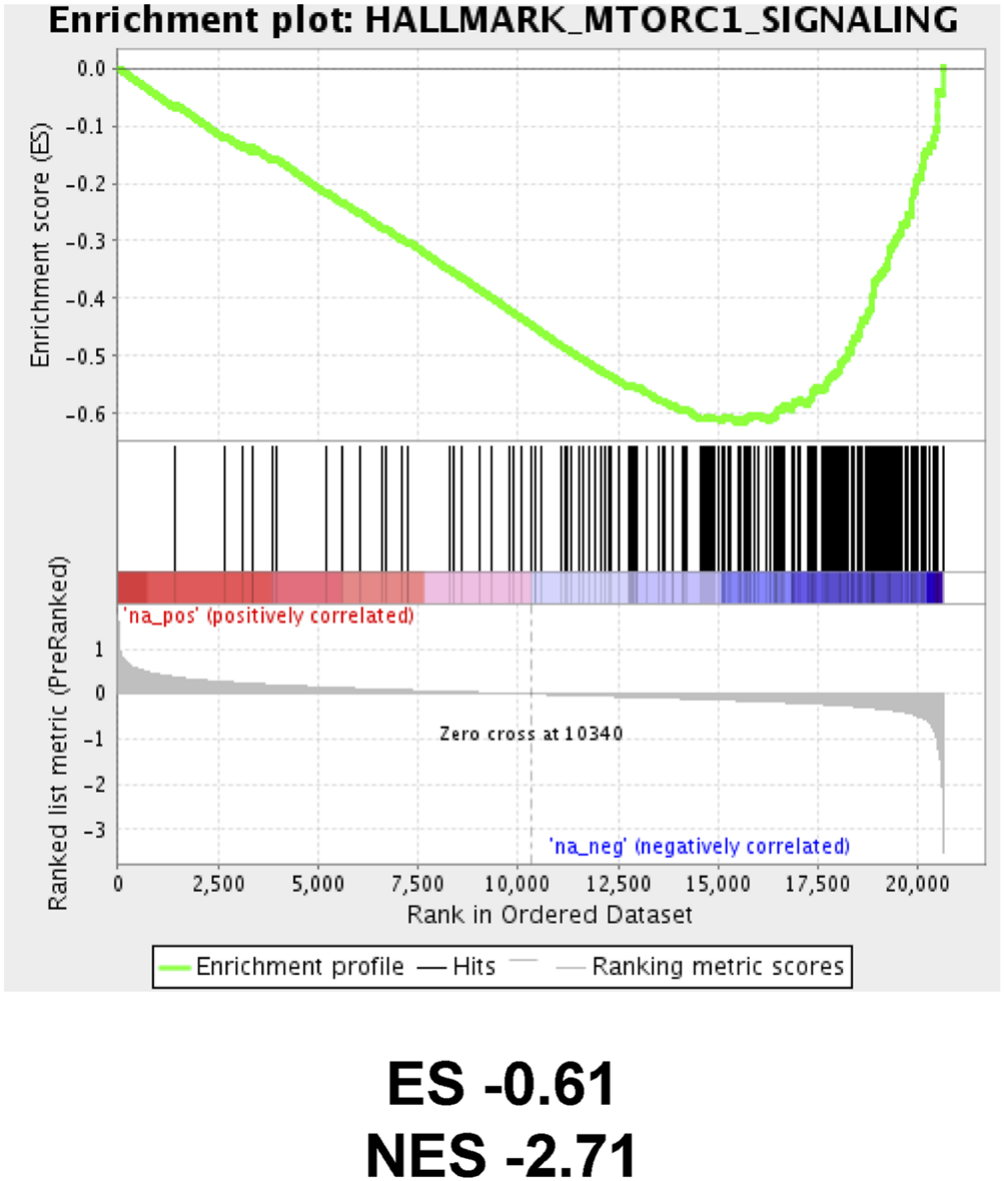
Suppression of mTOR signaling in breast cancer patients treated with abemaciclib. GSEA enrichment plot for mTOR signature following two weeks of abemaciclib monotherapy treatment (early vs baseline) in the neoMONARCH clinical trial. Nominal *p* < 0.01, FDR *q* < 0.01. ES, enrichment score; NES, normalized enrichment score; FDR, false discovery rate.

The inhibitory effects of abemaciclib on mTOR signaling in cancer cell lines and xenograft models all occur at drug concentrations that are readily achieved in patients receiving the recommended dose [2]. This implies that these effects may also be evident in patients. To test this prediction, we analyzed RNAseq data from a phase 2 study of neoadjuvant abemaciclib in postmenopausal, HR+, HER2- breast cancer patients (neoMONARCH, NCT02441946 [41]) and identified top scoring pathways via gene set enrichment analysis (GSEA). Following two weeks of treatment with abemaciclib monotherapy, in addition to cell cycle-related signatures (including G2M checkpoint and E2F targets [41]), the mTORC1 signaling pathway was significantly downregulated (Figure 7). A similar analysis of the publicly available dataset from the NeoPalAna study (NCT01723774 [42]) did not find mTOR signaling to be significantly altered following addition of palbociclib treatment for two weeks (data not shown). These results suggest that abemaciclib’s inhibition of the mTORC1 signature is not an indirect consequence of CDK4/6 inhibition and further support the conclusion that the mTOR pathway inhibitory activity of abemaciclib described in cancer cell lines also manifests in tumors from patients exposed to the drug.

## DISCUSSION

Previous studies of mTOR/S6 regulation by CDK4/6, Rb, and the cell cycle have yielded somewhat conflicting reports across various cellular contexts and require further investigation. S6 phosphorylation was shown to be temporally regulated during cell cycle progression, with lower levels of phosphorylation observed in G1 [43]. Negative regulation of TSC2 by exogenous cyclin D/CDK4/6 has been reported [13], and CDK4 or cyclin D1 knockdown led to a modest suppression of mTOR signaling in HER2+ breast cancer cells [9], effects which may contribute to the observed subtle reduction in S6 phosphorylation following long-term treatment with palbociclib (Supplementary Figure 1F) or CDK4/6 knockdown (Figure 1D). Conversely, long-term treatment of pancreatic cancer cells with palbociclib actually resulted in upregulation of mTOR signaling [44], and Rb loss or CDK4/ 6 inhibition activated mTORC2 and Akt in ovarian cancer, TNBC, and osteosarcoma cells [45], suggesting that the impact of CDK4/6 on mTOR signaling may vary by tumor type or genetic background.

Interpretation of *in vitro* biochemical and cellular assays evaluating targets of kinase inhibitors and translation to potential *in vivo* and clinical effects requires consideration of activities achievable at biologically relevant ATP concentrations and clinically relevant drug concentrations. For example, while abemaciclib displayed inhibitory activity against CDK9 in biochemical kinase assays (IC_50_ 57 ± 42 nM) [20], this activity did not translate to inhibition of CDK9 substrates in cells at circulating steady-state drug concentrations achieved in patients, or even at concentrations up to 20 µM [2, 46]. Inhibition of PIM kinases by abemaciclib was also identified through biochemical assays and was validated through the analysis of the PIM substrate BAD in cells [20]. In the present study, inhibition of mTOR signaling by abemaciclib was observed at concentrations < 1 µM in cells, which falls within the clinical exposure levels for the drug and its active metabolites [28], and thus is predicted to be potentially clinically relevant. Although clinical samples were not available to directly measure protein markers of pathway activation, such as S6 phosphorylation, analysis of the mTORC1 gene signature in RNAseq data from a phase 2 study of neoadjuvant abemaciclib (neoMONARCH, NCT02441946) revealed that abemaciclib monotherapy did indeed inhibit the mTOR pathway in HR+ breast cancer patients.

Though much work on PIM kinases has focused on their roles in hematological malignancies, with clinical testing of PIM inhibitors as monotherapy in multiple myeloma, acute myeloid leukemia, malignant lymphoma, and non-Hodgkin’s lymphoma [47], they have also been shown to be overexpressed in multiple solid tumor types, including breast, prostate, pancreatic, gastric, hepatocellular, and colorectal cancers [48–50] and hypothesized to contribute to disease progression in many cases. Across a large cell panel, the activity profiles of abemaciclib and palbociclib were closely related (*p* < 0.001), and *RB1* depletion conferred resistance to abemaciclib, suggesting that the predominant anti-tumor activity is driven by CDK4/6 inhibition [2]. Additionally, PIM447 was largely inactive as monotherapy across the cell panel (data not shown), suggesting that PIM inhibition alone will not confer significant anti-tumor activity. However, we note that in our prior study [2], there were a number of cancer cell lines in which the anti-proliferative IC_50_ of abemaciclib was at least 5× lower than palbociclib. Intriguingly, several of these, including MDA-MB-157 and HCC1143, are from triple negative breast cancer, which has been reported to depend on PIM kinase [51, 52]. Further work is necessary to determine whether PIM inhibition contributes to the diverging activity of the two drugs in these, or other, contexts and, more generally, to investigate the cancer types and context where combined CDK4/6 and PIM inhibition may be advantageous. In additional to their roles in cell growth and proliferation, PIM kinases have also been hypothesized to have immunosuppressive functions in cancer [53], in part via inhibition of JAK-STAT signaling [54, 55]. Whether these effects contribute to the ability of abemaciclib to promote anti-tumor immunity [56] remains to be evaluated.

PIM and CDK4/6 have each been implicated in resistance to PI3K inhibition [6, 15], while PI3K signaling can also contribute to CDK4/6 inhibitor resistance [57]. This crosstalk and the convergence of PIM and Akt on mTOR signaling led us to test the combination of BYL719 and abemaciclib in breast cancer cells, where we observed synergistic inhibition of cell growth and attenuation of downstream signaling. Dual inhibition of CDK4/6 and PIM by abemaciclib could present an advantage in combination with PI3K pathway inhibitors to suppress signaling and combat potential mechanisms of resistance to inhibitors of both PI3K and CDK4/6. PIM kinases have also been identified as mediators of drug resistance in other contexts [58], including resistance to MET inhibitors in lung or gastric cancers [59, 60], suggesting the application of PIM inhibition to other therapeutic combinations in additional tumor indications.

Several beneficial combinations of CDK4/6 inhibitors with compounds targeting PI3K/Akt/mTOR signaling have been reported in the preclinical setting [6, 7, 40, 57, 61–65]. Meanwhile, combinations of CDK4/6 inhibitors with PI3K/mTOR pathway inhibitors are at various stages of clinical testing, including abemaciclib with PI3K/mTOR inhibitor LY3023414 or everolimus; palbociclib with PI3K/mTOR inhibitor PF-05212384, PI3K inhibitors GDC-0077, taselisib, or pictilisib, or everolimus; and ribociclib with PI3K inhibitors alpelisib or buparlisib, or everolimus [18]. Ultimately, these studies will provide important information to understand the best strategies for combination therapy to take advantage of these complex biological relationships to improve clinical efficacy and circumvent resistance mechanisms.

## MATERIALS AND METHODS

### Cell lines and compounds

A549, BT-20, BT-474, BT-549, CAMA-1, DMS-53, DU4475, HCC38, HCC70, HCC202, HCC1143, HCC1187, HCC1395, HCC1569, HCC1806, HCC1937, HCC1954, HCC2218, HCT-116, Hs578T, Jeko-1, MCF-7, MDA-MB-134-VI, MDA-MB-157, MDA-MB-175-VII, MDA-MB-231, MDA-MB-361, MDA-MB-415, MDA-MB-436, MDA-MB-453, MDA-MB-468, MiaPaCa2, SK-BR-3, NCI-H441, SK-MEL-28, T-47D, U2OS, and ZR-75-1 were obtained from the American Type Culture Collection (ATCC) and cultured according to vendor recommendations. EFM-19 were obtained from the German Collection of Microorganisms and Cell Cultures (DSMZ) and SNU-886 were from the Korean Cell Line Bank (KCLB).

Abemaciclib, palbociclib, ribociclib, PIM447, BYL719, LY2090314, everolimus, DYRK1Bi AZ cpd 33 [32], dinaciclib, GSK2334470, abemaciclib metabolites M2 and M20 [28], and additional CDK4/6i (see Figure 2C [33],) were synthesized by Lilly Research Laboratories. AZD1208 (S7104) and additional palbociclib (S1579, see Supplementary Figure 1A) were purchased from Selleck Chemicals.

### Western blot analysis

Cells were treated as indicated and western blot analysis was performed as previously described [2]. The pS6 S235/236 (4858), pS6 S240/244 (2215), S6 (2317), 4EBP1 (9644), pRb S780 (8180), pRb S807/811 (8516), Rb (9309), pBAD S112 (5284), p-p70S6K T389 (9205), p70S6K (2708), PIM1 (3247), PIM2 (4730), PIM3 (4165), TSC2 (4308), pGSK3β S9 (5558), GSK3β (9315), pPRAS40 T246 (2997), PRAS40 (2691), and GAPDH (2118) primary antibodies were purchased from Cell Signaling Technology, while the CDK4 (ab75511) and CDK6 (ab124821) antibodies were from Abcam and β-actin (A5441) was from Sigma-Aldrich. IRDye secondary antibodies were from LI-COR.

### Cell growth assays

Cells were plated in 96-well plates and allowed to attach overnight prior to treatment with increasing concentrations of the indicated compounds. Cell growth was assessed by CellTiter-Glo^®^ Luminescent Cell Viability Assay (CTG; Promega), according to manufacturer’s instructions, or PI staining following treatment for two cell doubling times (2DT). PI staining was performed as previously described [46]. Response to drug combinations was evaluated by curve shift analysis [66] or Bliss independence analysis [67].

### *In vivo* study

All animal studies were performed in accordance with American Association for Laboratory Animal Care institutional guidelines, and all protocols were approved by the Eli Lilly and Company Animal Care and Use Committee. A549 cells (5 × 10^6^) were suspended in a 1:1 mixture of HBSS and Matrigel (BD Biosciences) and injected subcutaneously into the rear flank of female CB17SCID mice (Envigo). Mice were subsequently randomized into treatment groups (*n* = 5/group) based on tumor volume and body weight when mean tumor volume reached 200–250 mm^3^. A single dose (50 mg/kg) of abemaciclib or palbociclib was administered by oral gavage (PO) with tumors collected 2, 4, 8, or 24 h post-treatment. *In vivo* doses were selected in part based on previous pharmacokinetic data (data not shown), with 50 mg/kg palbociclib in mice corresponding to ~3× clinical exposures at 125 mg QD dose. Abemaciclib was formulated in 1% hydroxyethyl cellulose (HEC, Natrosol; Ashland) in 25 mM phosphate buffer, pH 2, while palbociclib was formulated in 5% N-methyl-2-pyrrolidone (NMP; Sigma-Aldrich), 0.05% antifoam in 25 mM phosphate buffer, pH 2. Vehicle 1% HEC in 25 mM phosphate buffer served as control. Tumors were homogenized and lysates were analyzed by western blot as described above.

### RNAi

Cells plated in 6-well plates were transfected with 25 nM *CDK4* (L-003238-00-0005), *CDK6* (L-003240-00-0005), *PIM1* (L-003923-00-0005), *PIM2* (L-005359-00-0005), *PIM3* (L-032287-00-0005), *TSC2* (L-003029-00-0005), or non-targeting control (D-001810-10-05) SMARTpool siRNA (Dharmacon) using DharmaFECT1 transfection reagent (T-2001-01, Dharmacon). Following 48 h transfection, cells were treated as indicated and/or lysed and analyzed by western blot.

### CRISPR *RB1* and *TSC2* knockout

DMS-53 and A549 cells were transfected with a plasmid containing Cas9 and *RB1*- or *TSC2*-specific sgRNAs, respectively, and subjected to puromycin selection (HD Biosciences, Shanghai, China). *RB1*, 5′CGGTGCCGGGGGTTCCGCGG 3′; *TSC2*, 5′ CTGTCGCACCATCAACGTCA 3′; 5′ CTGCAACTACCACGCTGCT 3′ (Sangon Biotech). For *RB1*, pooled KO cells were additionally subjected to selection with 2 µM palbociclib and *RB1* KO was confirmed by western blot. For *TSC2*, single clones were isolated and *TSC2* KO was validated by Sanger sequencing.

### Structural modeling

Preparation of the in-house PIM1 protein-ligand complex x-ray, the glide docking model and the superposition of palbociclib on top of abemaciclib were performed using the available Protein Preparation Wizard, Glide and manual atom pairs superposition panels of Maestro version 12.0.12 (Schrodinger Suites Release 2019-2).

An in-house PIM1 protein-ligand complex crystal structure where the ligand was structurally similar to abemaciclib (benzimidazole ring and fluorine atoms and their positions were conserved) was prepared. Waters were removed and not considered. A glide grid centered in the ligand and with defined two constrains to Glu121 (aromatic hydrogen bond) and Lys67 (H-bond) was built. This grid was used to dock abemaciclib 3D structure (taken from a published crystal structure of abemaciclib in CDK6, 5L2S. pdb) [24] using XP (Extra-precision) and rigid ligand docking (no sampling). The binding docking pose satisfied both constrains. The highest scored pose was selected and used to atom-pair superimpose palbociclib 3D structure (taken from 5L2I. pdb) [24] on top of it. The atom pairs list used was built based on the comparison between abemaciclib and palbociclib in previously cited crystal structures bound to CDK6.

### Clinical samples and GSEA

Postmenopausal early stage (Stage I [tumor ≥ 1 cm], II, IIIA or IIIB) HR+, HER2- breast cancer patients were randomized to one of three treatment arms: abemaciclib monotherapy (150 mg orally every 12 hours), anastrozole monotherapy (1 mg orally once daily), or abemaciclib in combination with anastrozole for two weeks, followed by an additional fourteen weeks of treatment with combination abemaciclib and anastrozole. Biopsies were obtained at baseline, following two weeks of initial therapy, and following the additional fourteen weeks of combination therapy. The study was conducted in compliance with the principles of good clinical practice, applicable laws and regulations, and the Declaration of Helsinki. The protocol was approved by each institution’s review board, and written informed consent was collected from patients before enrollment. This study is registered with ClinicalTrials. gov (NCT02441946). Extracted RNA from FFPE biopsy samples was analyzed by Illumina Truseq RNA exome RNA sequencing (RNAseq) and GSEA as previously described [41].

## SUPPLEMENTARY MATERIALS



## Abbreviations

CDK: cyclin-dependent kinase
SCLC: small cell lung cancer
MCL: mantle cell lymphoma
NSCLC: non-small cell lung cancer
TNBC: triple negative breast cancer
GSK3β: glycogen synthase kinase 3β
GSEA: gene set enrichment analysis
CTG: CellTiter-Glo^®^ Luminescent Cell Viability Assay
