# Supplementary material for: Combined inhibition of PIM and CDK4/6 suppresses both mTOR signaling and Rb phosphorylation and potentiates PI3K inhibition in cancer cells
Source: Oncotarget. 2020 Apr 28;11(17):1478–92. doi: 10.18632/oncotarget.27539 (PMC7197449; doi:10.18632/oncotarget.27539)
Supplement: Supplementary file 1 [file oncotarget-11-1478-s001.pdf]

Combined inhibition of PIM and CDK4/6 suppresses both mTOR signaling and Rb phosphorylation and potentiates PI3K inhibition in cancer cells

SUPPLEMENTARY MATERIALS

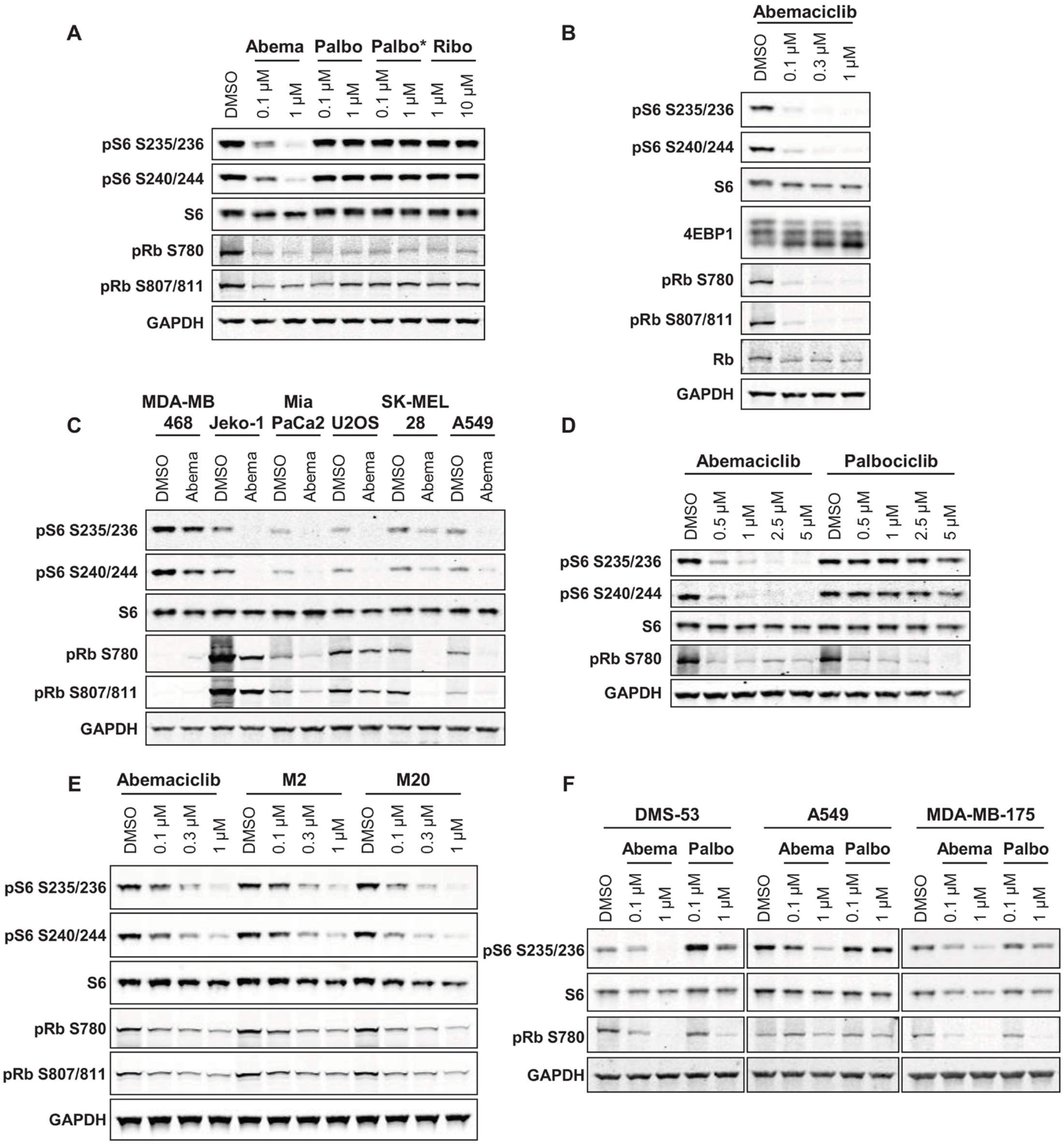

Supplementary Figure 1: Abemaciclib inhibits S6 phosphorylation. (**A**) DMS-53 cells were treated with abemaciclib, palbociclib, or ribociclib for 4 h and analyzed by western blot. *palbociclib from Selleck Chemicals (S1579). (**B**) DMS-53 cells were treated with abemaciclib for 24 h and analyzed by western blot. (**C**) The indicated cell lines were treated with 0.5 µM abemaciclib for 24 h and analyzed by western blot. (**D**) A549 cells were treated with abemaciclib or palbociclib for 24 h and analyzed by western blot. (**E**) DMS-53 cells were treated with abemaciclib or its metabolites M2 and M20 for 4 h and analyzed by western blot. (**F**) DMS-53, A549, and MDA-MB-175 cells were treated with abemaciclib or palbociclib for 2DT and analyzed by western blot.

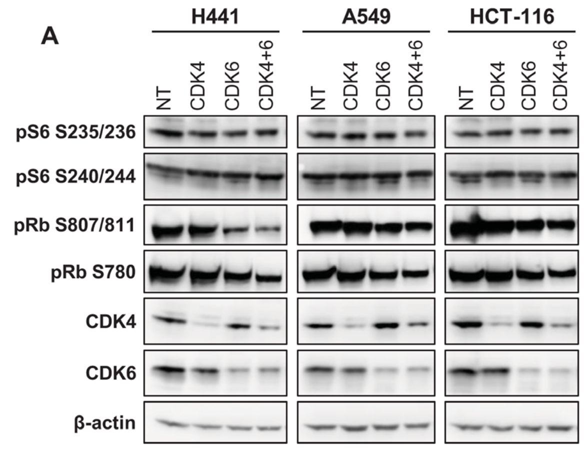

B

Kinase	%I @ 0.2 μM
PIM1	96.8
PIM2	74
PIM3	94.6

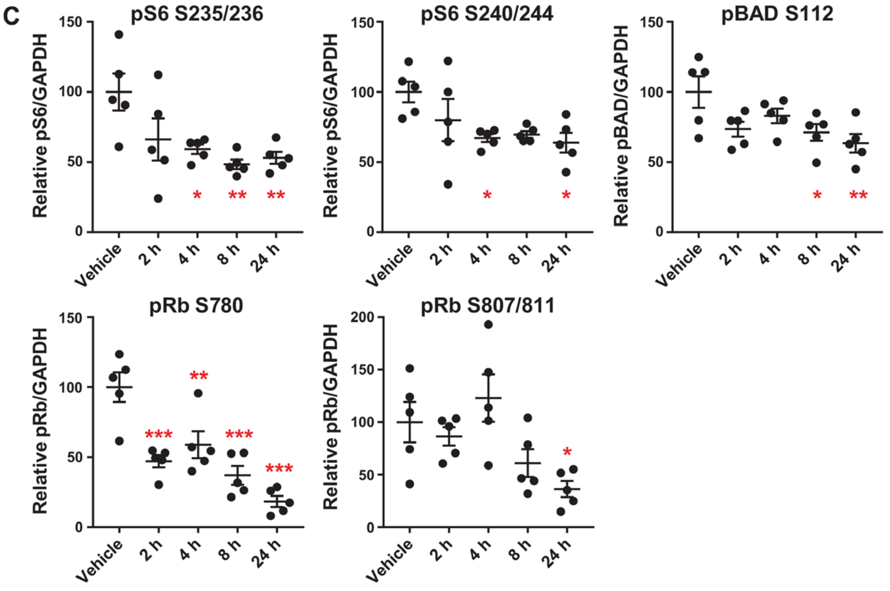

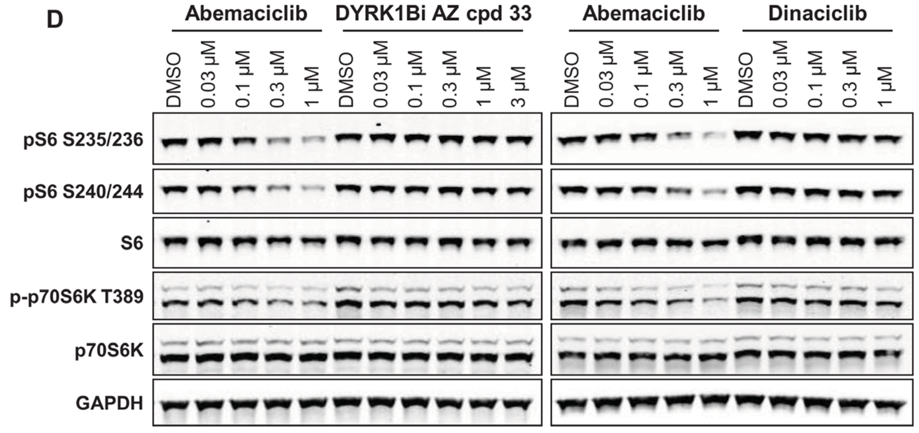

Supplementary Figure 2: CDK4/6, DYRK1B, and CDK9 inhibition does not alter S6 phosphorylation/mTOR signaling. (**A**) H441, A549, and HCT-116 cells were transfected with CDK4, CDK6, CDK4+CDK6, or non-targeting control (NT) siRNA and analyzed by western blot. (**B**) Inhibition of PIM kinases 1, 2, and 3 by abemaciclib via KINOMEscan analysis. (**C**) Mice bearing A549 xenograft tumors were treated with a single dose of abemaciclib (50 mg/kg). Tumors were collected 2, 4, 8, or 24 h post-treatment and analyzed by western blot. Plots indicate mean ± SEM (n = 5/group), relative to vehicle control. $^{*}p < 0.05$; $^{**}p < 0.01$; $^{***}p < 0.001$. (**D**) DMS-53 cells were treated with abemaciclib, DYRK1B inhibitor AZ cpd33, or dinaciclib for 4 h and analyzed by western blot.

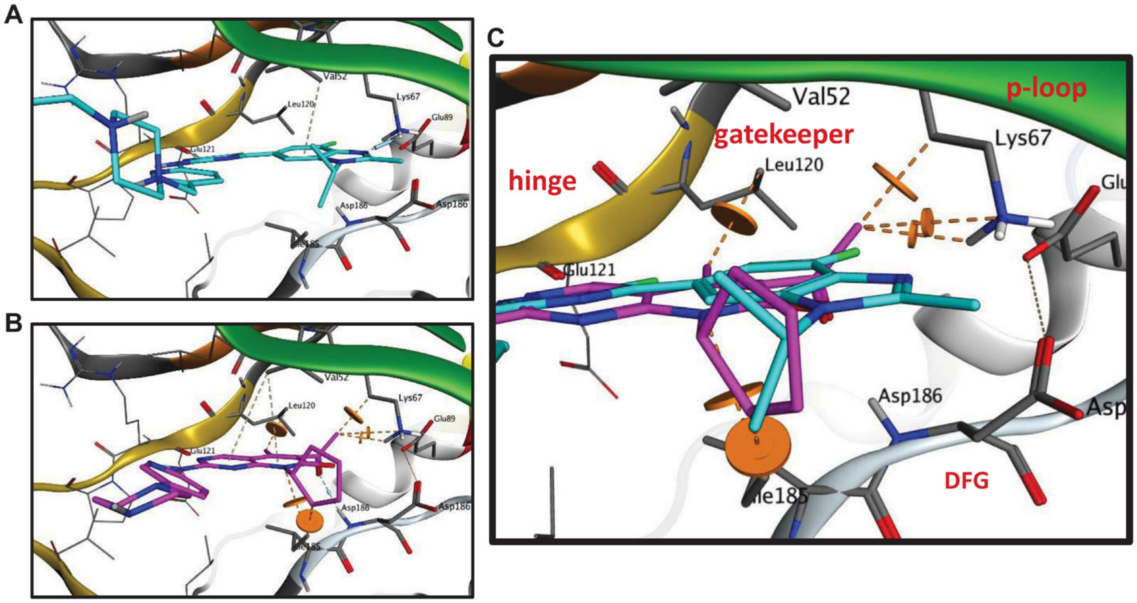

Supplementary Figure 3: Structural modeling of PIM1 with abemaciclib or palbociclib. (**A**) Abemaciclib predicted binding mode in ATP PIM1 pocket showed a hydrophobic interaction with Val52 in the p-loop represented by a gray dotted line and several polar H-bond interactions with Glu121 (hinge) and Lys67 (catalytic Lys) represented by blue dotted lines. (**B**) Palbociclib predicted binding pose showed multiple clashes inside the ATP PIM1 pocket represented by orange disks. (**C**) Comparison between abemaciclib and palbociclib in the equivalent binding mode (only clashes were represented).

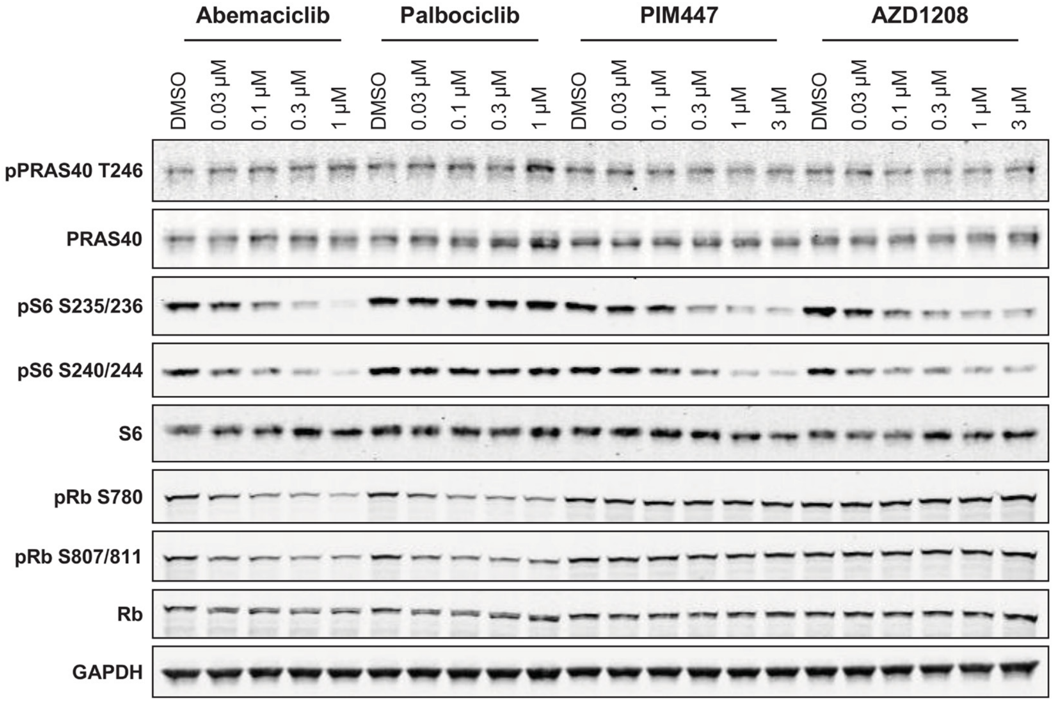

Supplementary Figure 4: Abemaciclib and PIM inhibitors do not alter PRAS40 phosphorylation. DMS-53 cells were treated with the indicated concentrations of abemaciclib, palbociclib, or PIM inhibitors PIM447 and AZD1208 for 4 h and analyzed by western blot.

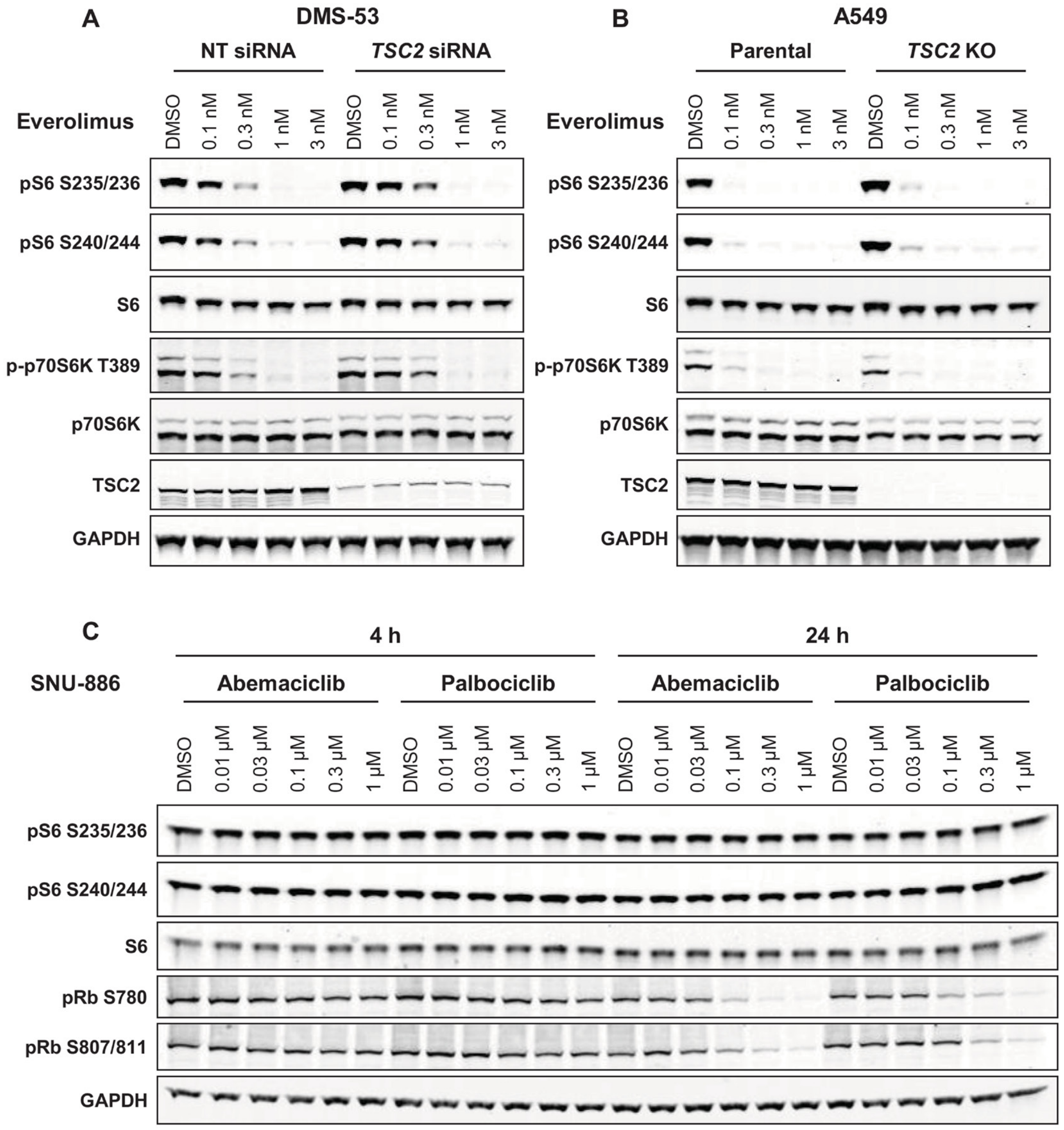

Supplementary Figure 5: *TSC2* is required for inhibition of S6 phosphorylation by abemaciclib but not by everolimus. (**A**) DMS-53 cells were transfected with *TSC2* or non-targeting control (NT) siRNA for 48 h, treated with everolimus for 4 h, and analyzed by western blot. (**B**) A549 parental or *TSC2* KO cells were treated and analyzed as in A. (**C**) *TSC2* mutant SNU-886 cells were treated with abemaciclib or palbociclib for 4 or 24 h and analyzed by western blot.

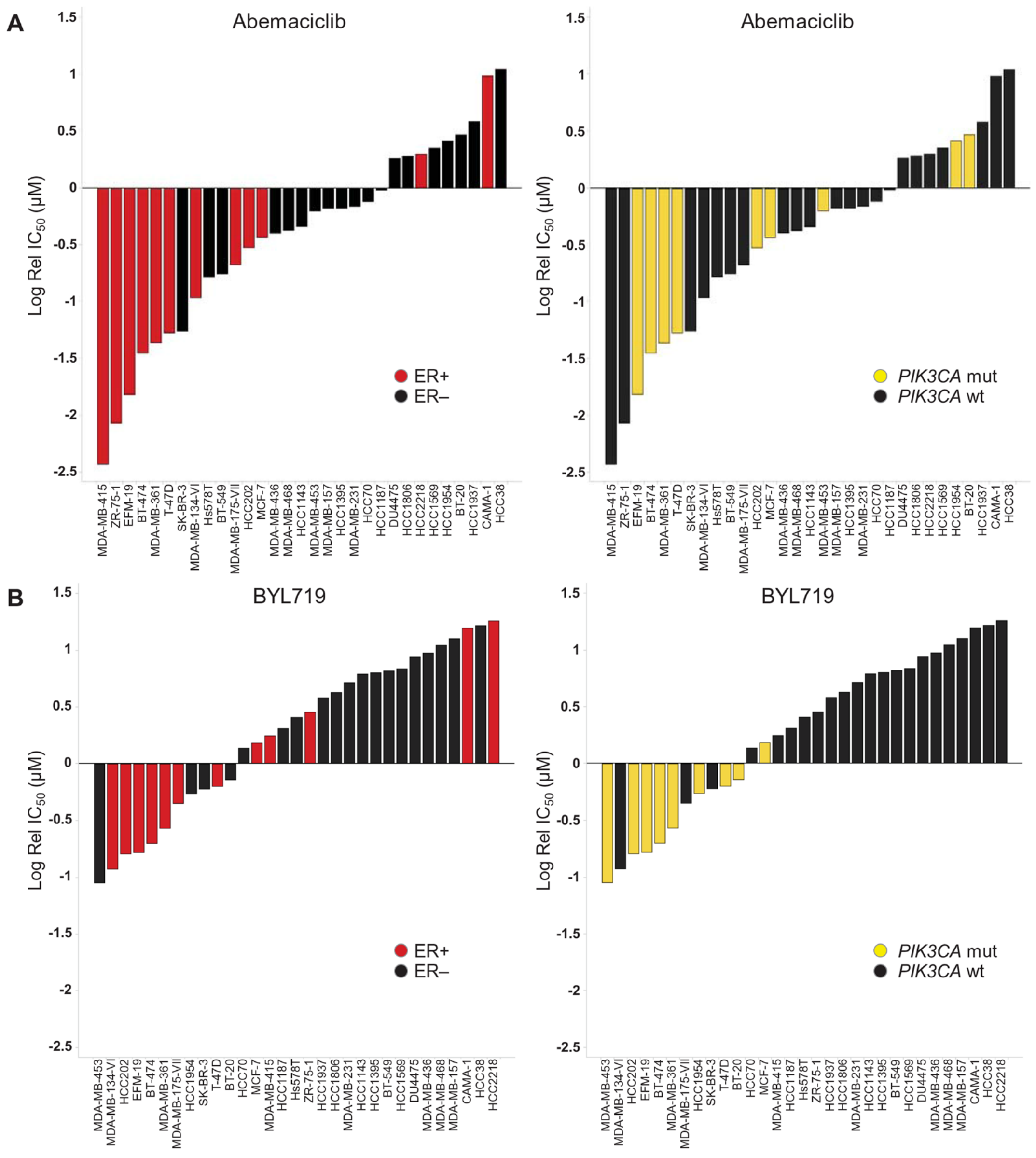

Supplementary Figure 6: Inhibition of breast cancer cell growth by single-agent abemaciclib or BYL719. A panel of 31 breast cancer cell lines was treated with single-agent abemaciclib (**A**) or PI3K inhibitor BYL719 (**B**) for 2DT and cell growth was assessed by PI staining. Log Rel IC_{50} (μM) values and ER expression/*PIK3CA* mutational status are displayed.

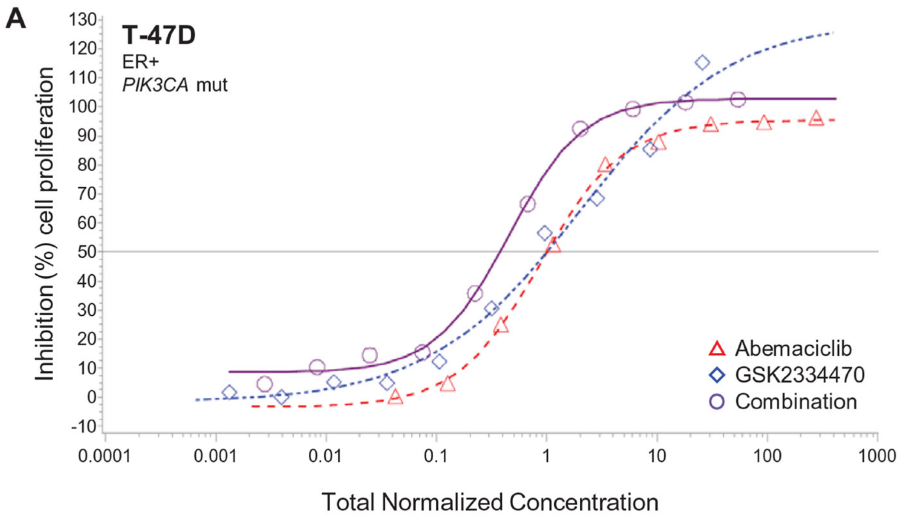

Cell Line	Combination Index
T-47D	0.377

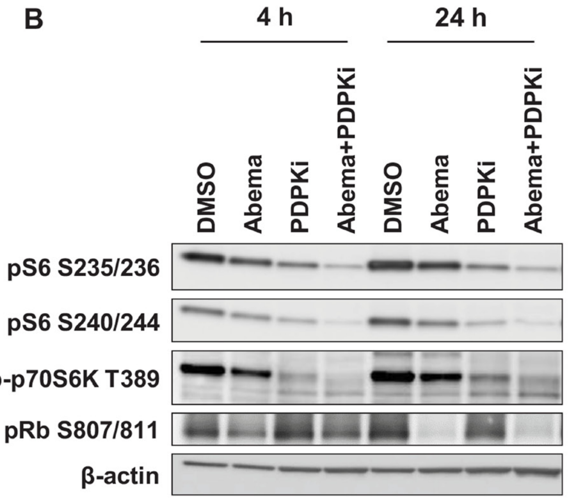

Supplementary Figure 7: Combination treatment with abemaciclib and PDPK inhibitor GSK2334470 synergistically inhibits breast cancer cell growth. (**A**) T-47D cells were treated with the combination of abemaciclib and GSK2334470 for 2DT and cell growth was assessed by PI staining. Curve shift analysis was used to calculate a combination index (CI) as an indication of additivity or synergy between the compounds. (**B**) T-47D cells were treated with the combination of abemaciclib (0.5 μM) and GSK2334470 (0.5 μM) for 4 or 24 h and analyzed by western blot.
